# Air stable and highly efficient Bi^3+^-doped Cs_2_SnCl_6_ for blue light-emitting diodes†

**DOI:** 10.1039/d1ra03622j

**Published:** 2021-08-02

**Authors:** Yue Yao, Si-Wei Zhang, Zijian Liu, Chun-Yun Wang, Ping Liu, Lan Ma, Guodan Wei, Feiyu Kang

**Affiliations:** Tsinghua-Berkeley Shenzhen Institute (TBSI), Tsinghua University Shenzhen 518055 China wangcy0317@gmail.com malan@sz.tsinghua.edu.cn weiguodan@sz.tsinghua.edu.cn; Tsinghua Shenzhen International Graduate School, Tsinghua University Shenzhen 518055 China

## Abstract

Cs_2_SnCl_6_ perovskite has recently attracted attention as a promising optoelectronic material owing to its better stability and reduced toxicity than its lead counterparts. However, its luminescence performance hardly satisfies the requirements. Hence, a series of Bi^3+^-doped Cs_2_SnCl_6_ (Cs_2_SnCl_6_:Bi^3+^) with enhanced luminescence were synthesized by a solution-phase route. The results show that the initial concentration of Sn^2+^ can adjust the nucleation density and the quality of the crystal nucleus growth, which will affect the Bi^3+^ doping amount, crystal morphology, and photophysical properties of Cs_2_SnCl_6_:Bi^3+^. Cs_2_SnCl_6_:Bi^3+^ shows excellent stability in the atmosphere with a photoluminescence (PL) of around 456 nm and a photoluminescence quantum yield (PLQY) of 31%. The luminescence performance results from [Bi_Sn^4+^_^3+^ + V_Cl_] defects caused by the Bi^3+^ doping. The blue LED based on the Cs_2_SnCl_6_:Bi^3+^ phosphor exhibits a long life of about 120 h and a Commission Internationale de L'Eclairage (CIE) color coordinates of (0.14, 0.11). This work demonstrates a strategy for Bi-doped perovskites with good stability. This investigation will facilitate the development of Cs_2_SnCl_6_:Bi^3+^ for blue LED applications.

## Abstract 2

A Bi^3+^-doped Cs_2_SnCl_6_ exhibits photoluminescence at around 456 nm and a photoluminescence quantum yield of 31%. The blue LED based on the Bi^3+^-doped Cs_2_SnCl_6_ phosphor exhibits a long life of 120 hours and a CIE color coordinates of (0.14, 0.11).

## Graphical abstract

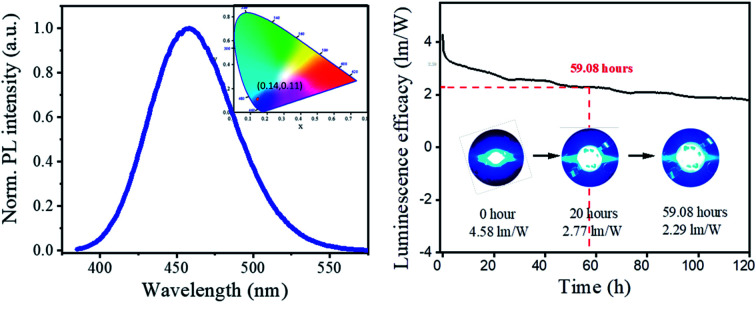

Lead halide perovskites have been extensively studied for their excellent performance in photovoltaics, light-emitting diodes (LEDs), and photodetectors. However, the toxic lead and dissatisfied stability of the lead halide perovskites suppress their practical applications, particularly the high solubility of Pb^2+^ in water, severely threatening the biological system and human health. Therefore, non-toxic Sn-based perovskites could be an ideal alternative.^1,2^ Sn^2+^-based CsSnCl_3_ barely shows any optoelectronic application due to its low PLQY, oxidation, and humidity,^3^ while Sn^4+^-based Cs_2_SnCl_6_ exhibits high anti-oxidation stability along with promising optoelectronic properties.^1^ Cs_2_SnCl_6_ has a vacancy-ordered double perovskite structure with isolated [SnCl_6_]^4−^ octahedra, improving the photoluminescence by quantum confinement effect.^4^

Several strategies have been reported to enhance the photoluminescence of Cs_2_SnCl_6_, and impurity doping has been demonstrated to be a facile and effective method.^2,4,5^ The defects in doped Cs_2_SnCl_6_ cause a charge imbalance, which must be rectified by the localization of electrons and holes or by the generation of impurity states within the bandgap, and this would add the localized level and affect luminescence properties.^6^ Numerous types of heteroatoms have been employed. Ce^3+^-doped Cs_2_SnCl_6_ could tune the [Ce_Sn^4+^_^3+^ + V_Cl_] defect density with different Ce^3+^ concentrations, affecting the crystal emission and exhibiting an achievable PLQY of 6.54%.^2^ Bi^3+^-doped Cs_2_SnCl_6_ (Cs_2_SnCl_6_:Bi^3+^) exhibited a higher PLQY of around 80% due to the hetero-valent replacement of Sn^4+^ by Bi^3+^ and exhibited a bandgap of about 3.05 eV,^4^ which was aspirational for the blue emitter. The defect density can also be affected by the doping process such as annealing temperature, subsequently changing the emission properties.^5^ The excellent Cs_2_SnCl_6_:Bi^3+^ shows great potential for practical applications. Cs_2_SnCl_6_ originates from Sn^4+^, but previous studies certified that only Sn^4+^ could not achieve the abundant doping of Bi^3+^. Although abundant studies have been carried out, the precursor concentration effects of Sn^2+^ and Sn^4+^ on the Bi^3+^ doping are still unknown, and the changes in morphology and photophysical properties caused by the doping process still need to be systematically reported. Simultaneously, the application of Cs_2_SnCl_6_:Bi^3+^ in LED devices, particularly the device stability, still needs more study.

In this study, Cs_2_SnCl_6_:Bi^3+^ was synthesized by a solution-phase route with varied precursor concentrations. The nucleation density and the quality of crystal nucleus growth can be tuned by the initial Sn^2+^ concentration, which can affect the crystal morphology, Bi^3+^ doping amount, and the photophysical properties of Cs_2_SnCl_6_:Bi^3+^. The nucleus growth process is accompanied by [Bi_Sn^4+^_^3+^ + V_Cl_] formation, which enhanced the luminescence intensity and extended the exciton delay time. The adjusted sample shows a PL peak of around 456 nm, PLQY of 31%, and good stability. More importantly, the blue LED based on Cs_2_SnCl_6_:Bi^3+^ phosphor exhibits a blue emission at the CIE coordinates of (0.14, 0.11), an external quantum efficiency (EQE) of 6.24%, and a luminescence power of 4.6 lm W^−1^. The device can continue to work for 120 h with blue luminescence.

The synthesis process of Cs_2_SnCl_6_ is shown in ESI.† In brief, two stages occurred as shown below. Sn^2+^ instead of Sn^4+^ was selected as the Sn precursor. By changing the amount of HCl added, the precursor solution's concentration (refers to the initial Sn^2+^ concentration) can be adjusted, affecting the nucleation density and quality of the crystal nucleus growth, then the morphology and photophysical properties of the product.

Stage 1: the nucleation processSnCl_2_ + 2HCl + O_2_ → SnCl_4_↓ + H_2_OSnCl_2_ + 2HCl → H[SnCl_3_]SnCl_4_ + 2HCl → H_2_[SnCl_6_]H_2_[SnCl_6_] + 2CsCl → Cs_2_SnCl_6_↓ + 2HClH[SnCl_3_] + CsCl → CsSnCl_3_↓ + HCl

Stage 2: the oxidation of CsSnCl_3_ and the further growth of Cs_2_SnCl_6_CsSnCl_3_ → CsCl + SnCl_2_SnCl_2_ + 2HCl + O_2_ → SnCl_4_↓ + H_2_OSnCl_4_ + 2HCl → H_2_[SnCl_6_]H_2_[SnCl_6_] + 2CsCl → Cs_2_SnCl_6_↓ + 2HCl

It can be concluded that Cs_2_SnCl_6_ originates from Sn^4+^, but previous studies certified that only Sn^4+^ cannot achieve the doping of Bi^3+^.^4^ Therefore, an Sn^4+^-poor/Sn^2+^-rich mixture was employed to facilitate the incorporation of Bi^3+^ into Cs_2_SnCl_6_. Therefore, SnCl_2_ was used as the precursor. It is still unclear at which step Bi^3+^ is doped. With the adjustment in the amount of HCl added, the concentration of Sn^2+^ in the precursor and Sn^4+^ produced in the reaction process can influence the Bi^3+^ doping. The amount and concentration of each reactant are listed in Table S1.† Monoclinic CsSnCl_3_ is observed when the precursor concentration of Sn^2+^ is too high (higher than 0.143 mol L^−1^), as shown in Fig. S1.† The monoclinic CsSnCl_3_ would significantly suppress luminescence and hinder Bi^3+^ doping. Consequently, the concentrations of the precursor of Sn^2+^ were investigated as 0.143, 0.125, 0.111, 0.100, and 0.091 mol L^−1^.

The scanning electron microscopy (SEM) images in Fig. 1 show the effects of the precursor concentration on the morphology of the Bi^3+^-doped Cs_2_SnCl_6_ (Cs_2_SnCl_6_:Bi^3+^) perovskite. The particles agglomerated to reduce the inherently high surface energy with a particle size less than 10 μm featuring phosphors' characteristics.^7^ Numerous small quasi-spherical particles adhered to the particles with high crystallinity, indicating that the crystal growth process is similar. The formation process of Cs_2_SnCl_6_ includes two stages: (1) the nucleation process of the initial Cs_2_SnCl_6_ and CsSnCl_3_ crystals and (2) the further growth of Cs_2_SnCl_6_ along with the oxidation of CsSnCl_3_. Both processes were related to the concentration of the initial reactants.

**Fig. 1:**
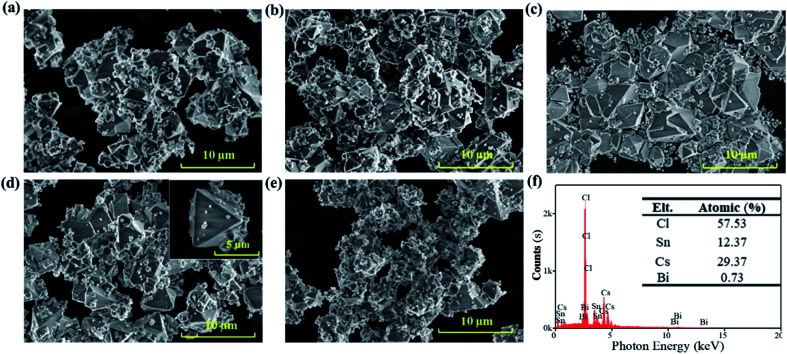
SEM images of Cs_2_SnCl_6_:Bi^3+^ synthesized at different precursor concentrations: (a) 0.143 mol L^−1^; (b) 0.125 mol L^−1^; (c) 0.111 mol L^−1^; (d) 0.100 mol L^−1^; (e) 0.091 mol L^−1^. (f) EDS results of Cs_2_SnCl_6_:Bi^3+^ obtained at 0.100 mol L^−1^.

High concentration of Sn^2+^ precursors (0.143 mol L^−1^ and 0.125 mol L^−1^) led to the abundant and dense nucleation of the initial Cs_2_SnCl_6_ and CsSnCl_3_ crystals in the first stage, consuming a considerable number of initial reactants. Therefore, there were not enough reactants for the continuation of the crystal growth, leading to some particles being agglomerated together without total growth (Fig. 1(a) and (b)). As the concentration decreased (0.111 mol L^−1^, 0.100 mol L^−1^), the nucleation of the initial Cs_2_SnCl_6_ and CsSnCl_3_ crystals decreased, and the crystals could grow fully. Fig. 1(c) and (d) show more large particles with good crystalline and clean surfaces, and the inset image of Fig. 1(d) shows the details of Cs_2_SnCl_6_ tetrahedral with thin particles. When the concentration decreased to 0.091 mol L^−1^, the second stage was severely suppressed due to the overdispersed nucleus. As a result, Fig. 1(e) shows only some small quasi-spherical particles with an insufficient crystal growth. Small-sized particles possessed higher chemical potentials, solubility and surface energy, which may not help improve the stability of Cs_2_SnCl_6_:Bi^3+^. Therefore, it can be concluded that the sample in Fig. 1(d) can exhibit higher stability. Further, the EDS of the sample at a concentration of 0.100 mol L^−1^ was characterized (Fig. 1(f)). 0.73% atomic Bi^3+^ was doped into the host material Cs_2_SnCl_6_, and the Bi^3+^-doped Cs_2_SnCl_6_ (Cs_2_SnCl_6_:Bi^3+^) was obtained.

The crystal structure properties of Cs_2_SnCl_6_:Bi^3+^ synthesized at different precursor concentrations are shown in Fig. 2(a). All of the XRD patterns are indexed to the Cs_2_SnCl_6_ phase (ICSD 29030, *Fm*3̄*m*, *a* = 10.369 Å),^8^ and no impurity phases can be observed, indicating its highly crystalline nature. However, the diffraction peaks show relatively small deviations when compared to that of the standard Cs_2_SnCl_6_ phase. The Rietveld refinement results show an enlargement of the lattice parameter within 0.41% for all the doped Cs_2_SnCl_6_ (Table 1 and Fig. S2†), which is ascribed to the different sizes between Bi^3+^ (1.01 Å) and Sn^4+^ (0.71 Å).^9^ More specifically, the strongest diffraction peaks that oriented towards the (220) direction demonstrate a left shift, as shown in the inset of Fig. 2(a), which implied the formation of chlorine vacancies [Bi_Sn^4+^_^3+^ + V_Cl_] when the Bi^3+^ cations randomly substituted Sn^4+^ cations.^2,4,5^

**Fig. 2:**
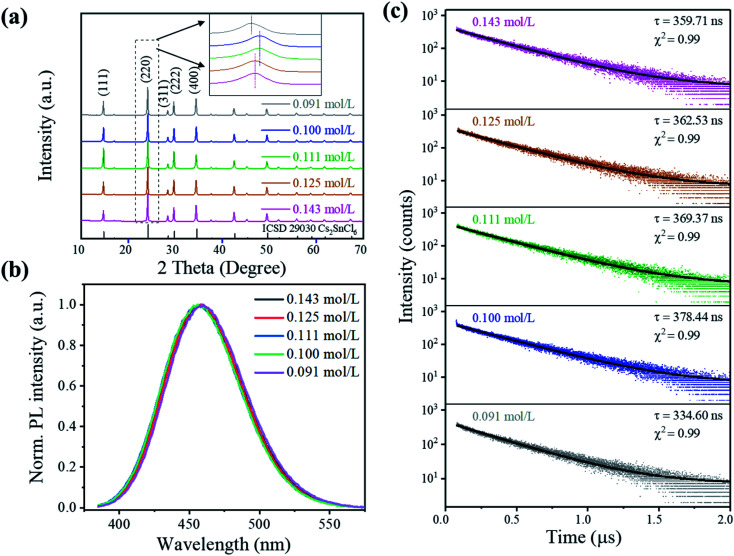
Structure and luminescence properties of Cs_2_SnCl_6_:Bi^3+^ synthesized at different precursor concentrations: (a) room-temperature XRD patterns and the enlarged (220) peak as an inset. (b) PL spectra of Cs_2_SnCl_6_:Bi^3+^ (*λ*_ex_ = 362 nm). (c) Time-resolved PL decay curve of Cs_2_SnCl_6_:Bi^3+^ (*λ*_ex_ = 375 nm and *λ*_em_ = 456 nm).

**Table 1:** The Rietveld refinement results of Cs_2_SnCl_6_:Bi^3+^ synthesized at different precursor concentrations (*Δ* = (*a* − *a*_0_)/*a*_0_ × 100%, *a*_0_ = 10.369 Å)^a^

Concentration (mol L^−1^)	*a* = *b* = *c* (Å)	*Δ* (%)	*R*_p_ (%)	*R*_wp_ (%)	*χ* ^2^
0.143	10.402	0.315	7.35	9.81	4.03
0.125	10.402	0.314	7.64	10.00	4.84
0.111	10.404	0.339	7.37	9.66	4.78
0.100	10.401	0.309	6.86	9.40	6.56
0.091	10.411	0.403	7.14	9.92	4.54

a *R*_p_: the reliability factor of the profile. *R*_wp_: weighted profile factor.

The evolution of the structure and change in morphology are inherently related. When the concentration gradually decreased from 0.143 to 0.100 mol L^−1^, the (220) peak demonstrated a right shift, indicating a smaller interlayer spacing caused by the [Bi_Sn^4+^_^3+^ + V_Cl_] formation. However, as the concentration decreases to 0.091 mol L^−1^, the (220) peak demonstrated a more significant left shift, which is attributed to a larger lattice parameter due to the larger ionic radius of Bi^3+^. The sample with 0.100 mol L^−1^ gained the highest density of [Bi_Sn^4+^_^3+^ + V_Cl_]. Simultaneously, the sample possessed the morphology of large particles, so it can be roughly recognized that the doping process of Bi^3+^ mainly occurred in the nucleation process (stage 1 mentioned above) and [Bi_Sn^4+^_^3+^ + V_Cl_] formed during the nucleus growth process with the consumption of Bi^3+^(stage 2).

The precursor concentration affects the morphology and phase composition, which further affected the perovskite's luminescence properties. Fig. 2(b) shows the PL spectra of the crystals under 362 nm excitation. The luminescence intensity of Cs_2_SnCl_6_:Bi^3+^ increases initially and reaches a maximum at 0.100 mol L^−1^, which rapidly decreases the PL intensity with the decrease in the concentration to 0.091 mol L^−1^, as shown in Fig. S5.† It could be attributed to the structural changes that were associated with [Bi_Sn^4+^_^3+^ + V_Cl_] formation, as no emission was observed from the undoped Cs_2_SnCl_6_.^4,5^ The excitation and emission peaks, full width at half maximum (FWHM), CIE color coordinates, and color purity luminescent materials are listed in Table S2.† All the samples showed a deep blue emission with an emission at around 456 nm and an FWHM of 65 nm. The color purity of Cs_2_SnCl_6_:Bi^3+^ is determined to be more than 90%, indicating that Cs_2_SnCl_6_:Bi^3+^ featured a higher color purity than the commercial blue-emitting compound BAM: Eu^2+^ (*C* = 88.0%).^10^ Since Cs_2_SnCl_6_:Bi^3+^ (0.100 mol L^−1^) showed the highest PL intensity among all the samples studied in this study, it was applied to the PLQY measurement with a PLQY value of 31%.

More insights into the luminescence mechanism are obtained from the time-resolved PL measurements, as shown in Fig. 2(c). The luminescence decay curve shows a single exponential decay behavior as evidenced by a linear curve when plotted on a logarithmic scale (*λ*_ex_ = 375 nm and *λ*_em_ = 456 nm) (see the formula in ESI†).^4,11,12^ Samples display a similar single-exponential luminescence decay behavior at different delay times, indicating that the concentration did not change the luminescence mechanism. The main part of the decay curve is fitted well by an exponential function. The decay time *τ* shows a tendency similar to PL intensity, as it extended from 359.71 ns (0.143 mol L^−1^) to 378.44 ns (0.100 mol L^−1^) and then shortened to 334.60 ns (0.091 mol L^−1^). Similar to MAPb (I_0.95_Br_0.05_)_3_ perovskite films reported by Wang *et al.*,^13^ the PL intensity of films increased as the proportion of the radiation recombination time *τ* increased, ascribing to the repairing of the deep-level defects of the perovskite surface.^13,14^

Wavelength-dependent PL and PL excitation spectrum (PLE) measurements were employed to study the luminescence mechanisms. As shown in Fig. 3(a), the excitation spectra show a redshift (≈6 nm), and the shape changes as the monitoring wavelength varies from 430 nm to 490 nm. The emission spectra are collected and shown in Fig. 3(b), which also show a shift (≈9 nm) as the exciting wavelength changes from 320 nm to 380 nm. In general, the doping-included ionoluminescence is vulnerable to the excitation energy. Furthermore, the change in the exciting energy leads to an apparent peak position shift and a shape change for photoluminescence because the excitation and the recombination rates depend primarily on the ion energy levels and their resonances.^4,15^ The possible state of dopant Bi in the Cs_2_SnCl_6_ host is [Bi_Sn_ + V_Cl_] instead of Bi^3+^ due to the broad excitation plateaus.^4,5^ The luminescence mechanism also proved that [Bi_Sn_ + V_Cl_] vacancies were introduced during the process when the Bi^3+^ cations randomly substituted the Sn^4+^ cations, consistent with the redshift of the (220) peak. Alternatively, [Bi_Sn_ + V_Cl_] is the primary source of Cs_2_SnCl_6_:Bi^3+^ luminescence, rather than Bi^3+^.

**Fig. 3:**
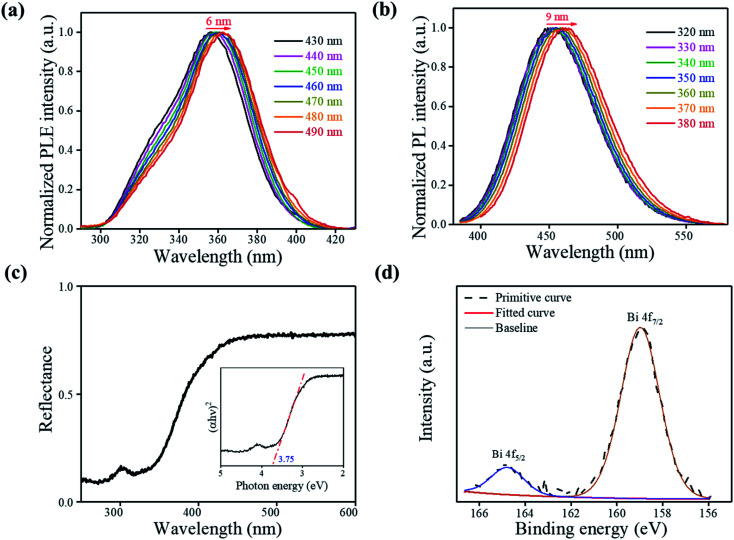
(a) Wavelength-dependent photoluminescence excitation spectrum. (b) Wavelength-dependent PL spectra. (c) Diffuse reflectance spectra (DRS). (d) High-resolution XPS spectra of Bi 4f of Cs_2_SnCl_6_:Bi^3+^ obtained at 0.100 mol L^−1^.

UV-Vis diffuse reflectance spectra (DRS) of Cs_2_SnCl_6_:Bi^3+^ were recorded to investigate the optical band gaps of the 0.100 mol L^−1^ sample, as shown in Fig. 3(c). The bandgap of Cs_2_SnCl_6_:Bi^3+^ can be estimated according to the following equation:^16^

1 [*F*(*R*_∞_)*hν*]^*n*^ = *A*(*hν* − *E*_g_)

where *hν*, *A* and *E*_g_ are the photon energy, proportional constant, and bandgap, respectively. For a direct bandgap of Cs_2_SnCl_6_, *n* = 2.^17^ From the linear extrapolation of function [*F*(*R*_∞_)*hν*]^*n*^ = 0, as shown in the inset of Fig. 3(c) (namely the Tauc plot),^11,16^ we can estimate the direct optical band gap to be 3.75 eV. The experimental value of *E*_g_ is lower than the theoretical value of 0.25 eV,^17^ such a shift of several nanometers could be attributed to the different defects associated with the absorption edge.^11^

X-ray photoelectron spectroscopy (XPS) measurement was carried out to further verify the product elemental composition. As shown in Fig. 3(d) and S3,† the XPS survey spectrum shows the characteristic peaks for Cs, Sn, Cl and Bi. The spectrum of Bi 4f exhibits Bi 4f_5/2_ and Bi 4f_7/2_ with binding energies of 164.8 eV and 159.0 eV, respectively, indicating Bi^3+^ is doped into the Cs_2_SnCl_6_ host material and form [BiCl_5_]^2−^ emission center (Fig. 3(d)). The peaks at 496.3 eV and 487.8 eV correspond to Sn^4+^ 3d_3/2_ and 3d_5/2_, respectively, proving that Sn^2+^ was oxidized entirely to Sn^4+^ after the reaction process in the reactor. The XPS spectroscopy directly proved the formation of [Bi_Sn_ + V_Cl_] defects and further verified the XRD pattern and luminescence mechanism.

The high quantum efficiency, good thermal stability, and blue emission of Cs_2_SnCl_6_:Bi^3+^ made it a promising candidate for blue-emitting LEDs. To demonstrate the application of Cs_2_SnCl_6_:Bi^3+^, a blue-emitting LED based on 365 nm UV-LED chips was fabricated. As shown in Fig. 4, the PL intensity increases gradually accompanied by an increase in current from 10 to 60 mA, and no apparent shift of the emission peak is found under different driving currents. The emission peaks were located at 460 nm, satisfying the commercial requirement of the blue light device (<480 nm).^18^ All of the spectra under different currents showed similar CIE chromaticity coordinates (0.14, 0.11), showing the high emission stability of Cs_2_SnCl_6_:Bi^3+^ blue LED.

**Fig. 4:**
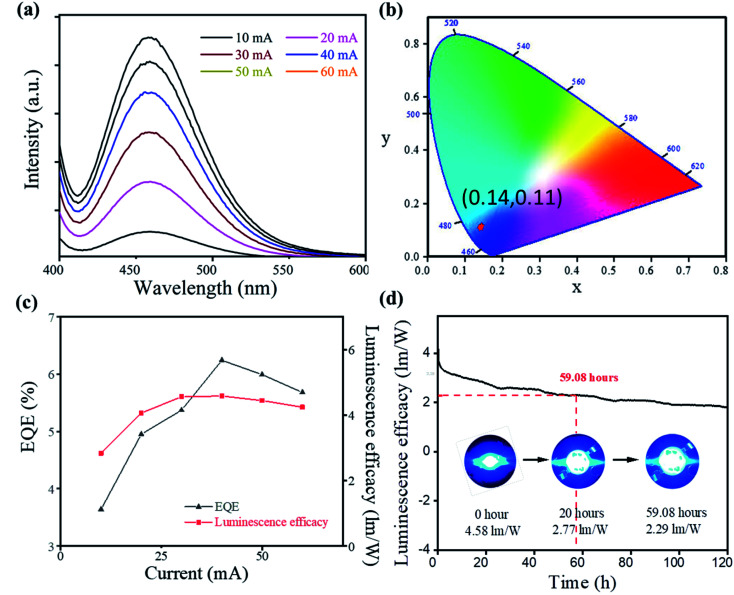
Blue LED device performance of Cs_2_SnCl_6_:Bi^3+^ (0.100 mol L^−1^): (a) the photoluminescence spectra at different currents. (b) CIE chromaticity coordinates of the blue LED. (c) EQE and luminescence efficacy at different currents. (d) Luminescence efficacy spectra of 120 h. Inset: photo images of the devices.

Furthermore, a maximum EQE of 6.24% and a luminous efficiency of 4.6 lm W^−1^ were obtained at 40 mA. More importantly, the device demonstrated an excellent stability, and time-dependent luminescence efficacy spectra appeared after 59.08 hours of continuous current. After 120 hour work, the device still showed good blue emission. The TGA measurement also showed a good thermal stability of the sample (Fig. S4†). The high thermal stability and the pure blue emission of Cs_2_SnCl_6_:Bi^3+^ made it a promising potential candidate for the blue-emitting LEDs.

A Bi^3+^-doped Cs_2_SnCl_6_ was synthesized *via* a solution-phase route with varied precursor concentrations. The nucleation density and the quality of crystal nucleus growth could be tuned by the precursor concentration, which could affect the crystal morphology, the Bi^3+^ doping amount, and the photophysical properties of Bi^3+^-doped Cs_2_SnCl_6_. The nucleus growth process was accompanied by [Bi_Sn_ + V_Cl_] formation, which enhanced the luminescence intensity and extended the exciton delay time. The larger crystallite size was synthesized at 0.100 mol L^−1^ concentration, with a highest PL intensity (456 nm, PLQY = 31%) and a most extended lifetime (378 ns) by suppressing the non-radiative process. Additionally, the samples demonstrated excellent stabilities, and the Bi^3+^-doped Cs_2_SnCl_6_ blue LED showed a blue emission with CIE coordinates of (0.14, 0.11), an external quantum efficiency (EQE) of 6.24%, and a luminescence power of 4.6 lm W^−1^. The device continued to work for 120 hours. This work suggested that Cs_2_SnCl_6_:Bi^3+^ possessed great potential in future light-emitting applications.

## Data statement

The data that support the findings of this study are available from the corresponding author upon reasonable request.

## Conflicts of interest

There are no conflicts of interest to declare.

## Supplementary Material

RA-011-D1RA03622J-s001
