# Supplementary material for: Air stable and highly efficient Bi3+-doped Cs2SnCl6 for blue light-emitting diodes
Source: RSC Adv. 2021 Aug 2;11(42):26415–20. doi: 10.1039/d1ra03622j (PMC9037418; doi:10.1039/d1ra03622j)
Supplement: RA-011-D1RA03622J-s001 [file RA-011-D1RA03622J-s001.pdf]

Electronic Supplementary Material (ESI) for RSC Advances.
This journal is © The Royal Society of Chemistry 2021

Air stable and high efficient Bi^{3+}-doped Cs_2SnCl_6 for blue light-emitting diodes

Yue Yao[1,2,#], Si-Wei Zhang,[1,#] Zijian Liu,[1] Chun-Yun Wang[1,2,*], Ping Liu[1,2], Lan Ma[2,*], Guodan Wei[1,2,*] and Feiyu Kang[1,2]

[1] Tsinghua-Berkeley Shenzhen Institute (TBSI), Tsinghua University, Shenzhen, 518055, China

[2] Tsinghua Shenzhen International Graduate School, Tsinghua University, Shenzhen, 518055, China

# These authors contributed equally to this work

Email: Chun-Yun Wang (wangcy0317@gmail.com)
Lan Ma (malan@sz.tsinghua.edu.cn)
Guodan Wei (weiguodan@sz.tsinghua.edu.cn)

Experiments

Synthesis of Cs_2SnCl_6:Bi: Cesium chloride (CsCl, Macklin, 99.99%); Tin (Ⅱ) chloride dehydrate ($SnCl_2{\cdot}2H_2O$, Macklin, 98%); Bismuth chloride ($BiCl_3$, Macklin, 99.99%); Hydrochloric acid (HCl, 37 wt% in water, Dongjiang Reagent Co., Ltd., China); Silica gel A and B (Shenzhen Looking Long Technology Co., Ltd., China). All chemicals were used without further purification. CsCl (673.4 mg, 4 mmol) and $BiCl_3$ (18.9 mg, 0.06 mmol) were added into 50 ml flask with different volumes HCl (9 ml, 11 ml, 13 ml, 15 ml, 17 ml), and stirring. The flask was loaded into an oil bath and heated to 80 °C for 2 mins. 451.3 mg (2 mmol) $SnCl_2{\cdot}2H_2O$ was dissolved in the 5 ml HCl, then heated to 80 °C for 1 hour. $SnCl_2{\cdot}2H_2O$ was added to the flask and left for 3 hours. After cooling to room temperature, samples were centrifuged (1000 rpm, 5 mins) and washed with ethanol three times. Sediment was dried at 50 °C in a vacuum oven for more than 12h.

Measurements: Structure, morphology and element composition of all samples were measured by X-ray Diffraction (XRD, D8 Advance, Cu Kα, λ=0.15418nm), Field-emission Scanning Electron Microscopy (SEM, HITACHI SU8010), X-ray Photoelectron Spectroscope with Co Kα radiation at a scan increment of 0.02°/min (XPS, PHI 5000V Versa Probe Ⅱ); PL, PLQY and scattering coefficients were

measured by PerkinElmer LS-55 fluorescence spectrometer (PL, continuous Xe lamp), FLS 980 spectrometer (PLQY, Edinburgh Instruments Ltd., UK). UV−vis spectra were recorded with a UV-3600 Plus spectrophotometer (Shimadzu Co. Ltd., Japan). All were measured at room temperature. Thermo-gravimetric analysis (TGA) and differential scanning calorimetry (DSC) was carried out at a temperature range of 25-700 °C (NETZSCH STA 449F3 STA449F3A-0672-M).

Fabrication of blue LED devices: Mixing silica gel A and B and sample in a mass ratio of 1:4:1.3, then adding this mixture on the top of a 365 nm UV LED chip(Shenzhen looking long technology co., LTD) with a luminescence efficacy of 1.12 lm/W at 40 mA.

Table. S1 Reaction Conditions of different volume of HCl

Volume (ml)	CsCl (mmol)	$BiCl_3$ (mmol)	$SnCl_2$*$2H_2O$ (mmol)	Precursor concentration* (mol/L)
10	4	0.06	2	0.200
12	4	0.06	2	0.167
14	4	0.06	2	0.143
16	4	0.06	2	0.125
18	4	0.06	2	0.111
20	4	0.06	2	0.100
22	4	0.06	2	0.091

* The precursor concentration refers to the initial concentration of the Sn^{2+}.

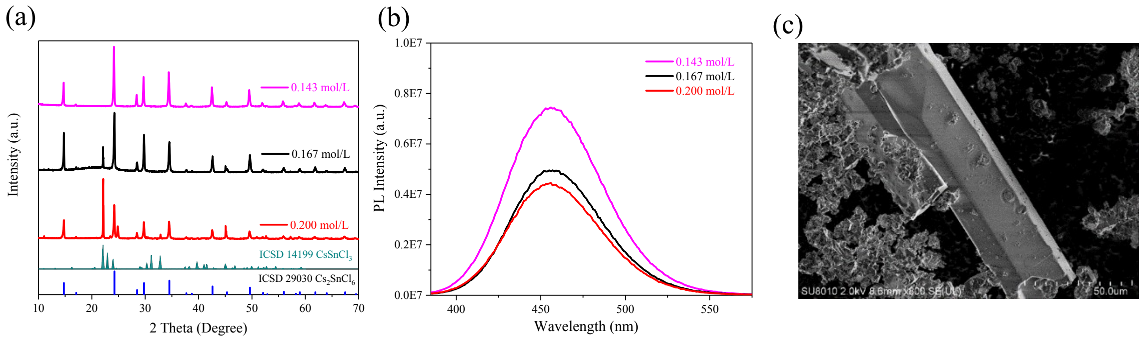

FIG. S1. Measurement results of sample synthesized at 0.200, 0.167, 0.143 mol/L: a) XRD patterns; b) PL spectra at same measurement condition (λ_{ex}=362 nm). (c) SEM image of sample synthesized at 0.200 mol/L

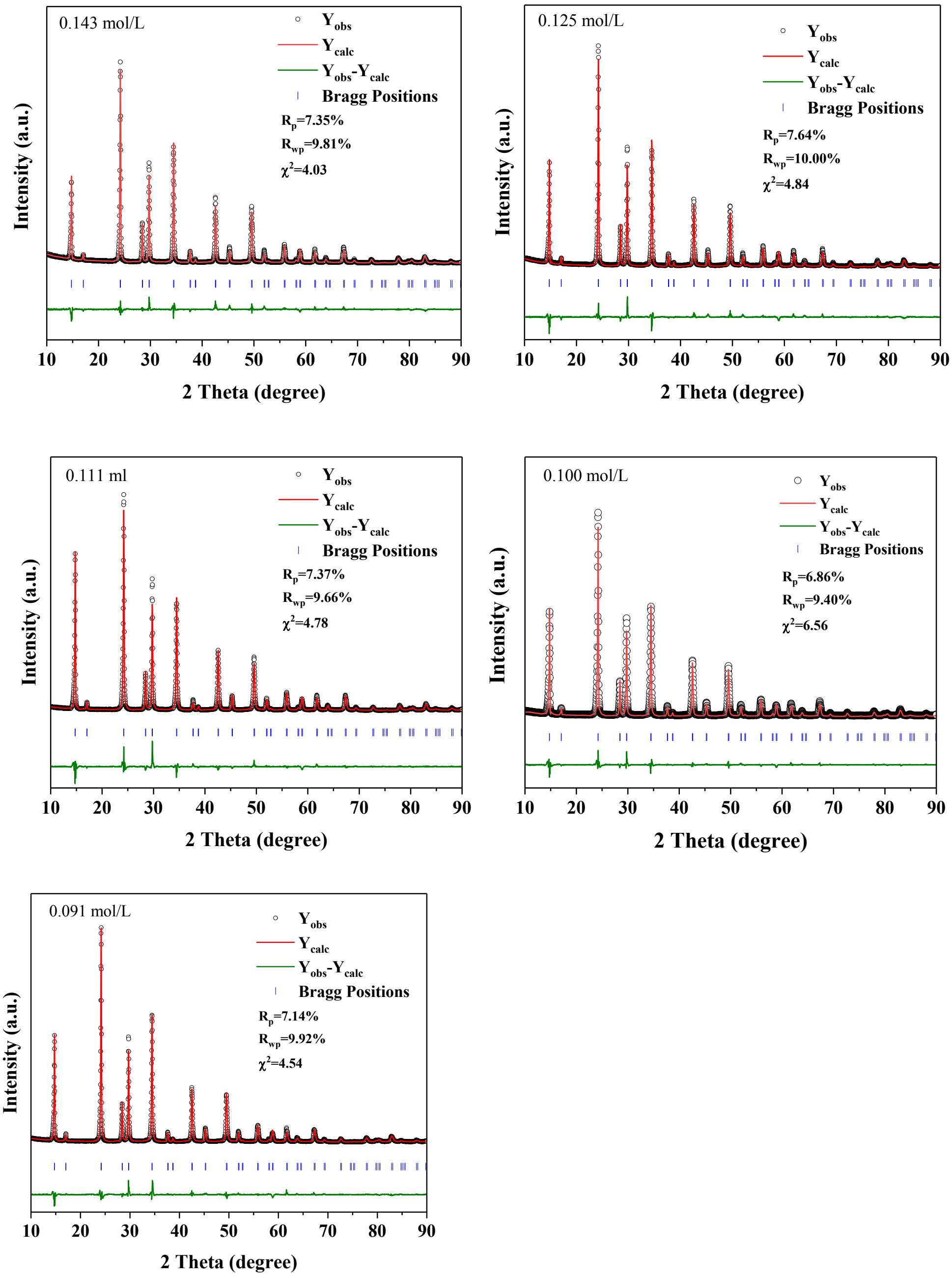

FIG. S2. The Rietveld refinements XRD patterns of Cs_2SnCl_6:Bi^{3+} synthesized at different precursor concentration

Table S2 The details of PL spectra of $Cs_2SnCl_6:Bi^{3+}$ synthesized at different solvent volume

Con. (mol/L)	Emission peak (nm)	Excitation Peak (nm)	FWHM (nm)	CIE (x, y)		C (%)
				x	y	
0.143	456	362	65.19	0.140	0.085	91.58
0.125	456	362	65.43	0.140	0.085	91.57
0.111	457	362	65.08	0.140	0.090	90.01
0.100	456	362	65.71	0.139	0.091	90.33
0.091	459	362	65.88	0.138	0.100	90.51

The following formula was employed to judge the color purity of $Cs_2SnCl_6:Bi^{3+}$ as this parameter is an important feature to evaluate phosphor chromaticity property:[2]

$$C = \frac{\sqrt[2]{(x - x_i)^2 + (y - y_i)^2}}{\sqrt[2]{(x_d - x_i)^2 + (y_d - y_i)^2}} \times 100\% \qquad (1)$$

where C is color purity; (x, y) and (x_d, y_d) is the CIE chromaticity coordinates of luminescent material and emission peak, respectively. (x_i, y_i) is CIE coordinates.

The room-temperature PL decay curve of the samples was measured and fitted by the equation (2):[3]

$$I = A \exp(-t/\tau) \qquad (2)$$

Where I represent the PL intensity of the sample as a function of time; A is a constant; t is time, and τ denotes the decay time.

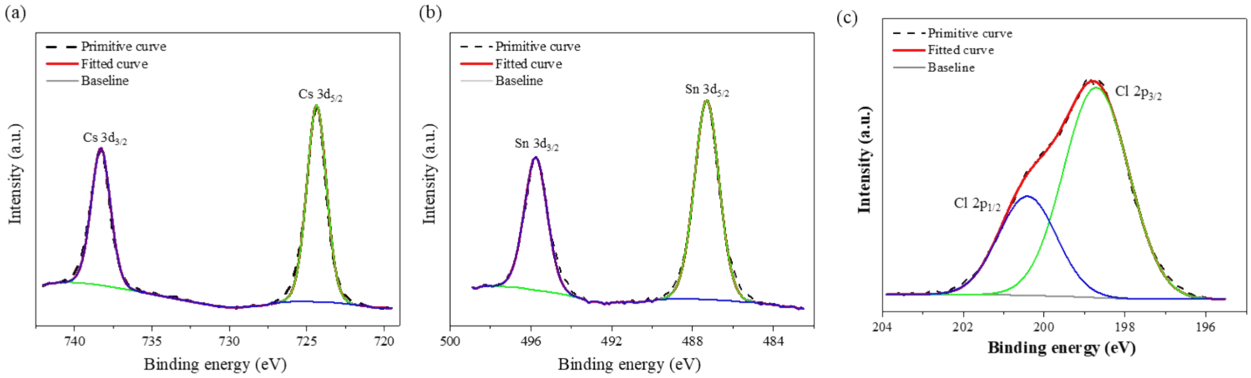

FIG. S3. The XPS data of $Cs_2SnCl_6:Bi^{3+}$ obtained at 0.100 mol/L

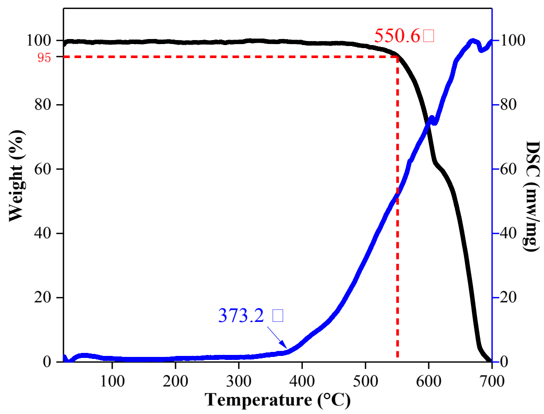

FIG. S4. TGA and DSC spectra of $Cs_2SnCl_6:Bi^{3+}$ obtained at 0.100 mol/L

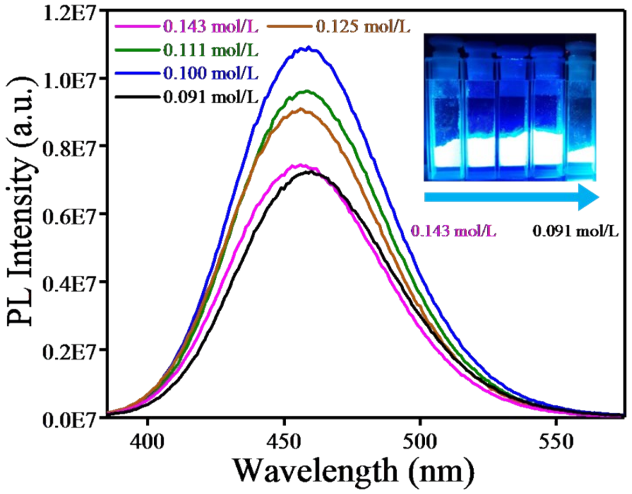

FIG. S5. PL spectra of $Cs_2SnCl_6:Bi^{3+}$ (λ_{ex} = 362 nm) and the photo image (inserted).

Thermo-gravimetric analysis (TGA) and differential scanning calorimetry (DSC) in flowing N_2 at a heating rate of 1 °C/min were carried out to analyze thermal stability of synthetic $Cs_2SnCl_6:Bi^{3+}$. The thermal decomposition of $Cs_2SnCl_6:Bi^{3+}$ starts at 373.2 °C, and the extrapolated onset temperature is 366 °C. The weight decreases to 95% at the high temperature of 550.6 °C. It indicates $Cs_2SnCl_6:Bi^{3+}$ has inherent thermal stability compared with the typical ABX_3 halide perovskites and organic-inorganic hybrid perovskite (such as $CH_3NH_3PbCl_3$).[4]

[1] H. Zhang, L. Zhu, J. Cheng, L. Chen, C. Liu, and S. Yuan, Crystals. **9**, 258 (2019).

[2] J. Zheng, Q. Cheng, S. Wu, Z. Guo, Y. Zhuang, Y. Lu, Y. Li, and C. Chen, Journal of Materials Chemistry C. **3**, 11219 (2015).

[3] Z. Tan, J. Li, C. Zhang, Z. Li, Q. Hu, Z. Xiao, T. Kamiya, H. Hosono, G. Niu, E. Lifshitz, Y. Cheng, and J. Tang, Adv. Funct. Mater. **28**, 1801131 (2018).

[4] A. Dualeh, P. Gao, S. I. Seok, M. K. Nazeeruddin, and M. Grätzel, Chem. Mater. **26**, 6160 (2014).
